# Cerebrospinal fluid: a rising subject in brain development

**DOI:** 10.3389/fnmol.2013.00030

**Published:** 2013-10-04

**Authors:** Angel Gato

**Affiliations:** Anatomy Department, Universidad de ValladolidValladolid, Spain

**Keywords:** commentary, brain, development, spondin, CSF

Many research studies in developmental neurosciences have been focused on neuroepithelial cellular events at the tissue, cellular, and molecular levels, in order to understand how the brain develops from a single structure, the neural tube. Although a couple of researchers (such as Mary Desmond, from Villanova University as an outstanding figure) introduced the idea that the cavity of the neural tube and its content, the embryonic cerebrospinal fluid (CSF), plays a key role in the brain development, it is not until the last two decades, with the work of several research groups that embryonic CSF has been considered an essential part of the developing brain, which exerts a direct influence on basic neuroepithelial behavior such as cellular survival, replication, and neurogenic differentiation.

The CSF composition during embryogenesis and fetal period is really complex as has been shown by proteomic analysis. However, we are yet to determine the final composition of this fluid. In this way the paper of Vera et al. is relevant because they report the presence of new components in the developing CSF, such as SCO-Spondin with a specific effect in neurogenesis. This paper also establishes a link between embryonic CSF research and the research focused in circumventricular organs (specially the Subcommissural Organ) secretion and biological role in which Prof. Esteban Rodriguez is a relevant figure.

In addition, this paper supports the idea that CSF composition and biological properties change and evolve across development with the addition of specific components in a temporally and spatially regulated manner, giving us the idea that there are several aspects of neural precursors behavior control, regulated by CSF which changes progressively from embryo to the adult.

Finally this paper is relevant because it is focused on a specific component of CSF involved in a local regulation of neurogenesis suggesting that this fluid can play global but also local, regulatory roles in developing brain.

